# An efficient protocol to use iron oxide nanoparticles in microfluidic paper device for arsenic detection

**DOI:** 10.1016/j.mex.2018.10.017

**Published:** 2018-10-27

**Authors:** Shraddha Chauhan, Lata Sheo Bachan Upadhyay

**Affiliations:** Department of Biotechnology, National Institute of Technology Raipur, Raipur, Chhattishgarh, 492010, India

**Keywords:** Colorimetric microdetection of arsenic with μPAD, μPAD, Arsine, Arsenic, Iron oxide nanoparticles

## Abstract

This method describes a rapid ecofriendly and affordable method for detecting arsenic in the water sample. The system designed works on the principle that involves generation of arsine due to reduction of arsenic by bare and cysteine capped iron oxide nanoparticles and its further reaction with silver nitrate present on the microfluidic paper analytical device (μPAD). Change in the color of μPAD from colorless to reddish brown is a result of reaction between arsine gas and silver nitrate, and is the detection criteria. The sample solution of arsenic was prepared in lemon juice to provide the required acidic environment for hydride generation. This proposed method has detection limit of 0.01 ppm (10 ppb) and 1 ppm for cysteine capped and bare iron oxide nanoparticles respectively. This is for the first time that iron oxide nanoparticles are being used for detection and reduction arsenic species in environmental sample. The same device can be used for on-site detection in an ecofriendly manner.

## Graphical abstract

**Figure:**
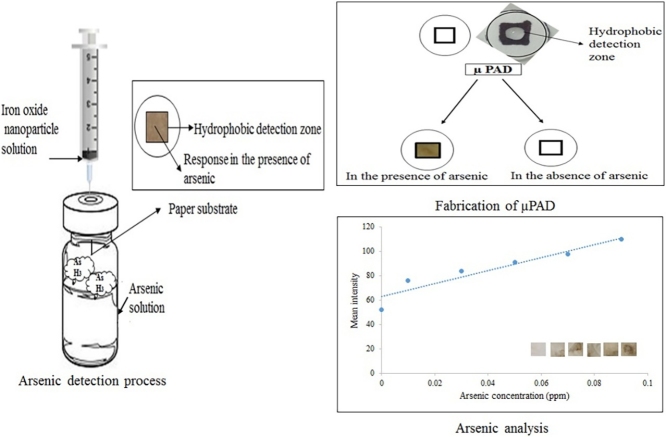

**Specifications Table**

**Table:** 

Subject Area	Environmental Science
More specific subject area:	Water quality assessment
Protocol name:	Colorimetric microdetection of arsenic with μPAD
Reagents/tools:	All chemicals used in this experiment were of analytical grade. The solutions were prepared in deionized water (MQ) from Milipore^®^ (Merck KGaA Darmstadt, Germany). The stock of arsenic was prepared using arsenic trioxide salt (As_2_O_3_). μPAD was prepared by applying / coating silver nitrate (AgNO_3_) solution on whatman filter paper 1 as arsine recognizing element. Fresh lemon juice was used for the generation of free hydride in the reaction mixture. 1 M sodium hydroxide (NaOH) was used for stabilizing the pH of the nanoparticle synthesis mixture. Whatman filter paper No. 1 was used from Whatman (GE Healthcare Life science). A camlin permanent marker pen was used to create the hydrophobic barrier on the paper. The reaction was carried out in empty injection vials collected from All India Institutes of Medical Sciences (AIIMS) Raipur. Piranha solution was prepared by mixing sulfuric acid (H_2_SO_4_) and hydrogen peroxide (H_2_O_2_) and was used to clean the discarded vials collected from AIIMS hospital. Green tea (procured from Organic India) and ferric chloride (FeCl_3_) and cysteine were used for iron oxide nanoparticle synthesis. All the chemicals used were purchased from Loba Chemie (Mumbai, India).
*Experimental design:	Bare and cysteine capped Iron oxide nanoparticles as reducing agents was injected into the clean injection vial containing 1 ml of the arsenic salt contaminated sample. The reaction between the nanoparticle and arsenic ion takes place in an aqueous phase under acidic environment. Nanoparticle mediated reduction of arsenic ion is resulted into formation of/ release of arsine gas (AsH_3_) in the reaction vial. The inner top of the vial cap was fitted with circular Whatman filter paper disk 1.0 cm (diameter) supplemented with 20 μl of 5% AgNO_3_ solution. The released arsine gas finally react with the AgNO_3_ on the filter paper to develop/give reddish brown color. The arsine silver nitrate complex give rise to change in the color of the filter paper and intensity of the developed color depends on the concentration of arsenic species in the sample. The intensity of the color was a measure of the amount of arsenic present in the sample [1].
Trial registration:	–
Ethics:	No ethical issues involve
*Value of the Protocol:	• A low cost instrumental free method for arsenic analysis is proposed. • The method gives colorimetric reaction that can be useful for the qualitative assessment of the water with respect to arsenic contamination. • The method is very quick and easy, it includes organic reagents that do not cause any toxic effect to the environment. • The assay can be used as a vanguard analytical system for the determination of arsenic.

## Description of protocol

**Fig. 1:**
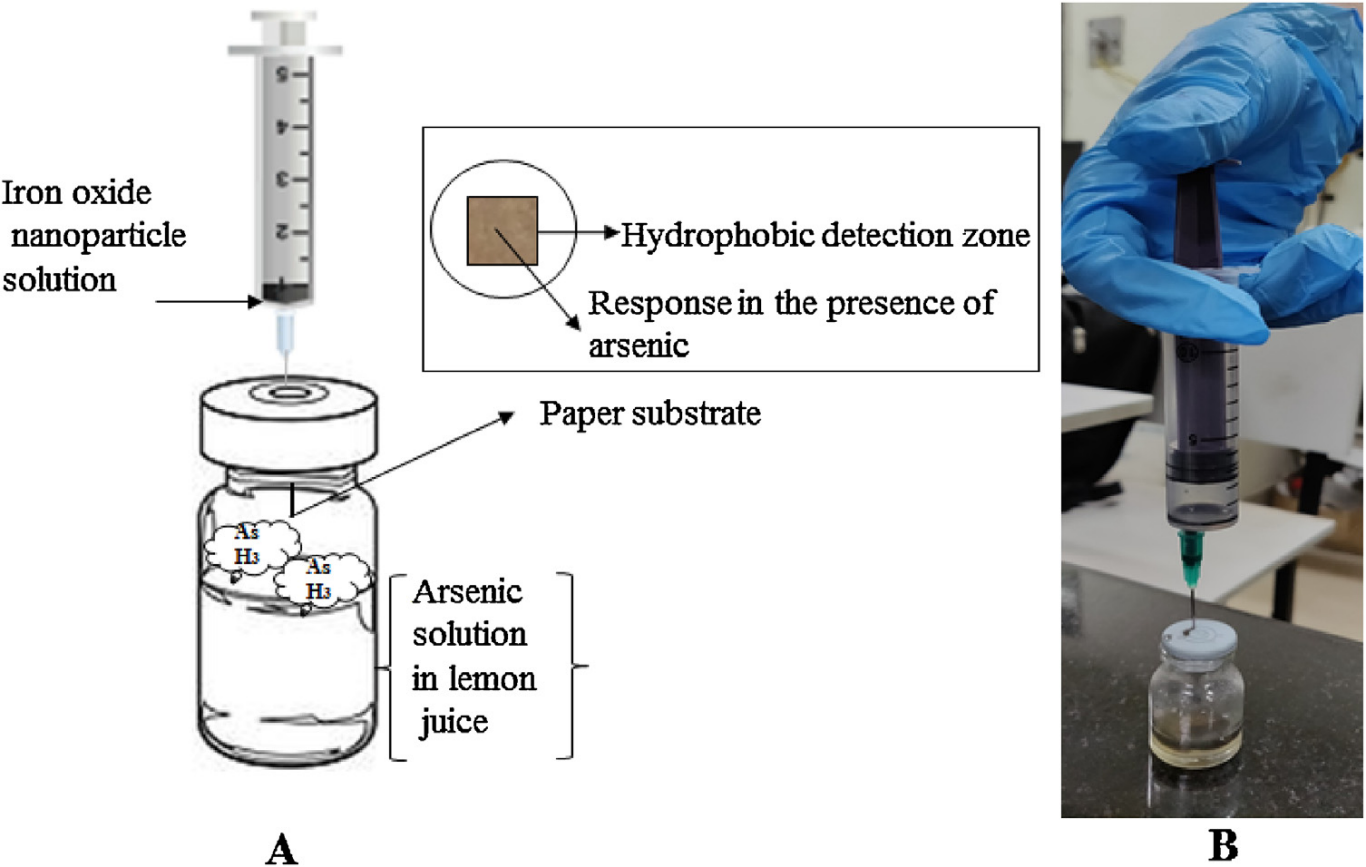
A) Schematic representation of the process for arsenic detection B) Experimental setup.

**Fig. 2:**
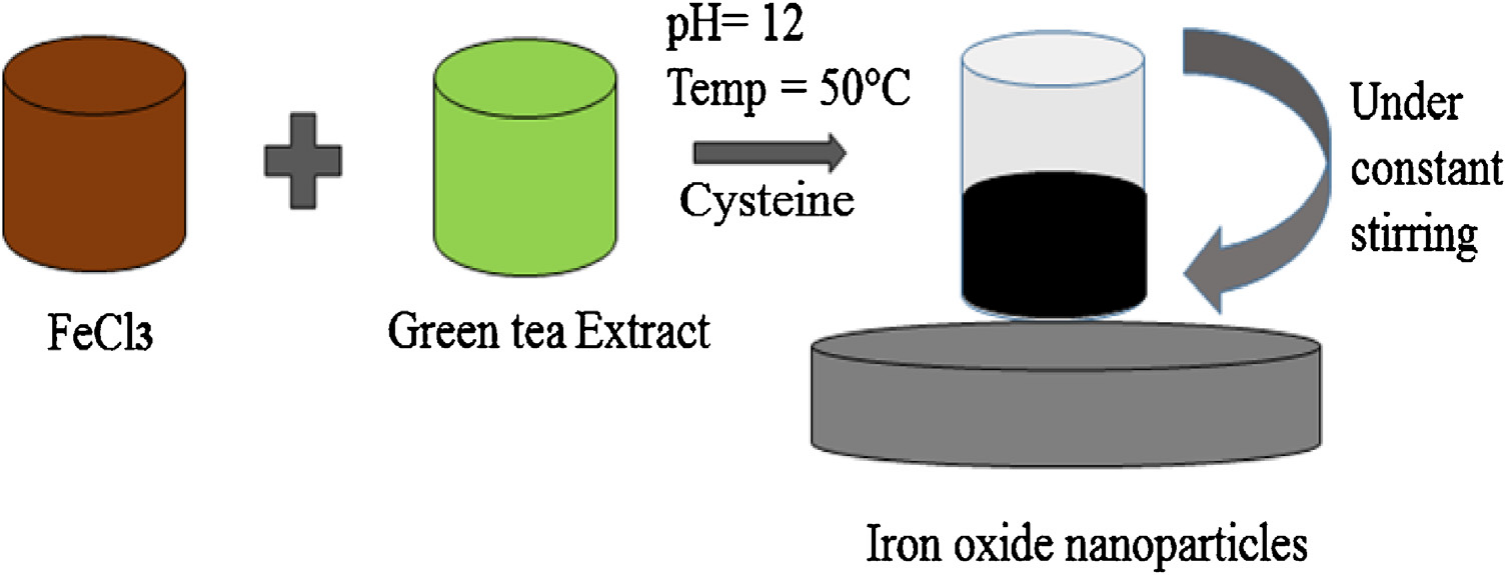
Green synthesis of iron oxide nanoparticles.

The schematic representation of the process is shown in Fig. 1 and details are as follows:

• **Cleaning of the injection vials:** The discarded injection vials were used in this process. These vials were first washed with piranha solution and then autoclaved to clean the impurities/contaminants. Piranha solution was prepared by mixing H_2_SO_4_ and H_2_O_2_ in 3:1 ratio.
• **Preparation of arsenic stock:** The acidity of the solution places an important role in the selective generation of hydrides from arsenic. For that purpose the lemon juice was used to provide the acidic environment to the procedure 10 mg of As_2_O_3_ was mixed with 10 mL of lemon juice this solution was taken as the standard arsenic stock solution for the analysis throughout the experiments.
• **Synthesis of cysteine capped and bare iron oxide nanoparticles:** Iron oxide nanoparticles were used in this process as it has reported to have strong reducing ability. Iron oxide nanoparticle’s synthesis was performed employing green technology, green tea extract was mixed with 0.01 M FeCl_3_ salt solution in 1:1 ratio (v/v), For capped nanoparticle synthesis 0.02 M cysteine was used during the synthesis process to provide the capping of the iron oxide nanoparticles. Green tea extract cause reduction of FeCl_3_ into iron oxide nanoparticles by the action of polyphenolic compound present in the extract. The iron salt reduction immediately gives a black color solution that indicates the presence of iron oxide nanoparticles in the aqueous mixture. The process is represented in Fig. 2 shown below.

**Fig. 3:**
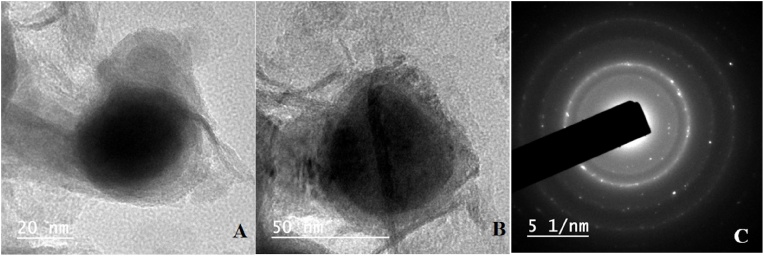
TEM images of cysteine functionalized iron oxide nanoparticles A)-(C) with scale bars of 20 nm and 50 nm.

**Fig. 4:**
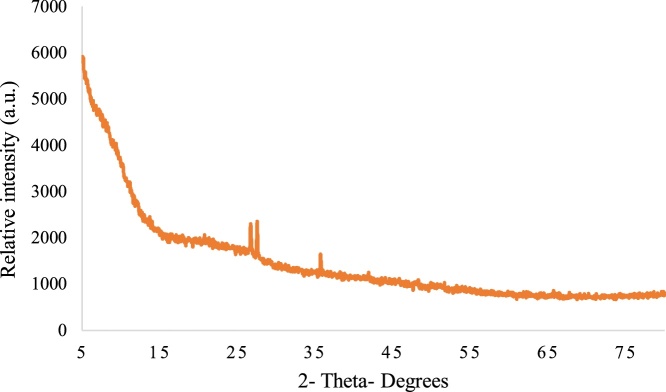
XRD pattern of green synthesized nanoparticle.

The synthesized cysteine capped iron oxide nanoparticles were characterized through TEM and XRD. For the study of the morphology and structure of the iron oxide nanoparticle’s TEM analysis was carried out. Fig. 3 indicates that the synthesized nanoparticles are of spherical shape and have a size range of 20–50 nm. Further XRD analysis of the prepared nanoparticles were done for better understanding of the structure of prepared nanoparticles. All the patterns observed are deficient in characteristic diffraction peaks, that reflects that the nanoparticles are amorphous in nature with high purity (Fig. 4).

**Fig. 5:**
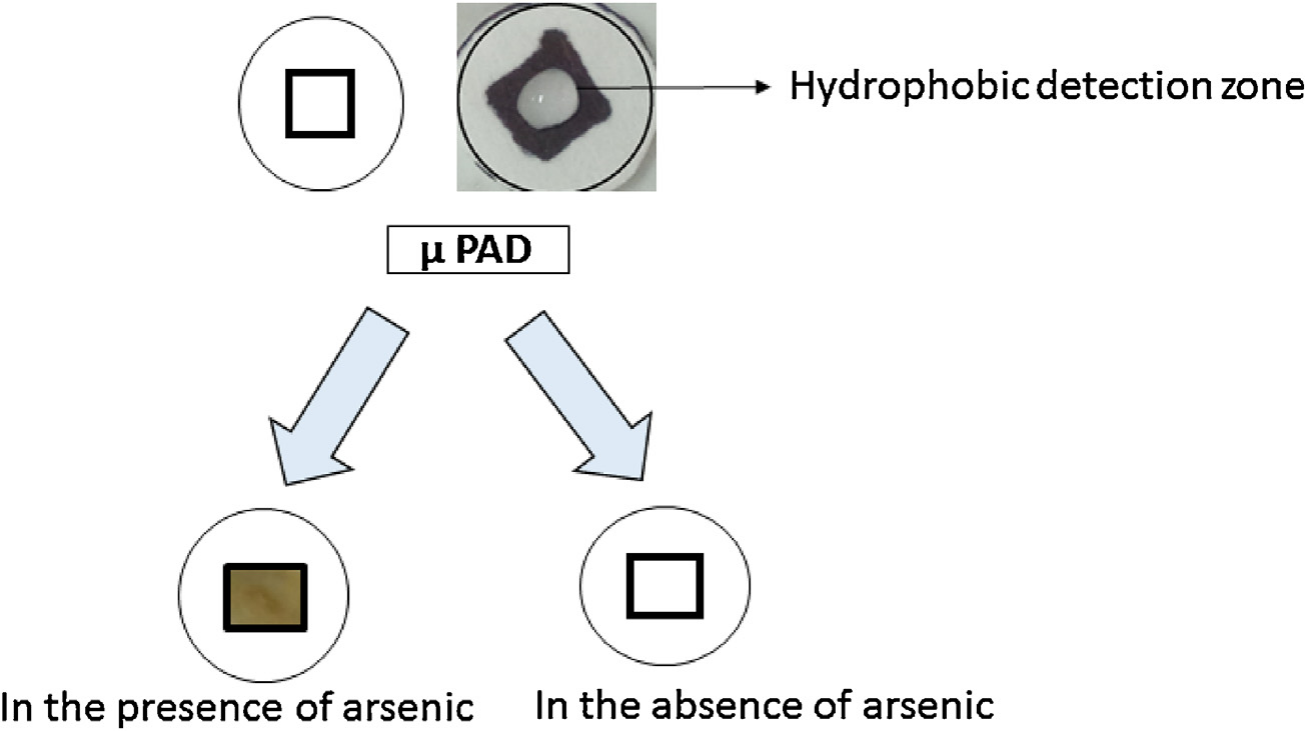
Fabrication of μPAD and its response towards arsenic.
• **Analysis of the arsenic:** The prepared μPAD was placed on the inner surface of the rubber cap of the glass vials with help of double tape to secure the position of the μPAD at the center of the rubber cap. The same arrangement can also be done in vials with screw hole cap over a PTEF faced septum. AgNO_3_ that act as a recognition element for arsine gas was exposed on the top of the vials for analysis. The determination of arsenic concentration was done by placing 1mL of arsenic solution in 10 mL injection vials. The arsine was generated in the vials after adding 0.5 mL of bare and capped iron oxide nanoparticles solution (Fig. 1). The vials were lightly shake to provide the mixing. The reaction mixture was incubated at room temperature for 10 min. Presence of arsenic in the sample was indicated and detected by development of reddish brown color on the μPAD due to formation of silver arsine complex. On completion of incubation period, the colored silver nitrate-impregnated filter paper placed in the vial cap was removed for image capturing and analysis. A corresponding blank was also run in absence of iron oxide nanoparticles. The arsenic detection analysis was performed for both lab samples as well as environmental water sample. The river water sample was collected from Kharun river of Raipur, Chhattisgarh. The concentration of total arsenic was analyzed by using this method with cysteine capped iron oxide nanoparticles and arsenic was found to be present at 0.68 ppm concentration. To validate the results obtained from this method, the same sample was analyzed from atomic absorption spectroscopy. The results of analysis are displayed in Table 1.

• **Fabrication of a μPAD:** The design of a paper based microfluidic device is based on a simple pattern plotting method [2]. The paper was cut into circular disk of 1 cm diameter. The hydrophobic zone was created with the help of camlin permanent marker pen by making a square of 0.5 (l x b) on both sides of disk. The paper was then allowed to air dry for 5 min and placed in a hot air oven at 70 °C for 1 h. oven treatment of the filter paper was to evaporate ink solvent from the paper surface so that an inert hydrophobic zone can be created (Fig. 5). This hydrophobic zone is used as a detection zone in this experiment. The oven dried paper was spotted with 20 μL of 5% AgNO_3_ solution in the hydrophobic detection zone prepared on the PAD. The AgNO_3_ spotted paper was again dried under dark condition and stored in amber color air tight zipper bag/pouch at −20 °C till further use.

**Table 1:** Analysis of water.

Mode of analysis of Arsenic concentration	AAS	0.7 ppm
	μPAD	0.68 ppm
• **Evaluation of the color mode:** The color mode was detected by the software imageJ this gives the mean intensity of the color zone generated in the paper. The higher concentration gives higher mean intensity values. The paper substrate were first scanned with desktop scanner, these digitalized images were processed by imageJ. The images were converted to RGB color mode and the mean intensity of blank and samples were analyzed (Fig. 6). In red green blue channels red color mean intensity was selected for analysis of the process as it provides the lowest mean intensity. The method was applied to determine total arsenic present in the sample as shown in Fig. 6.

**Fig. 6:**
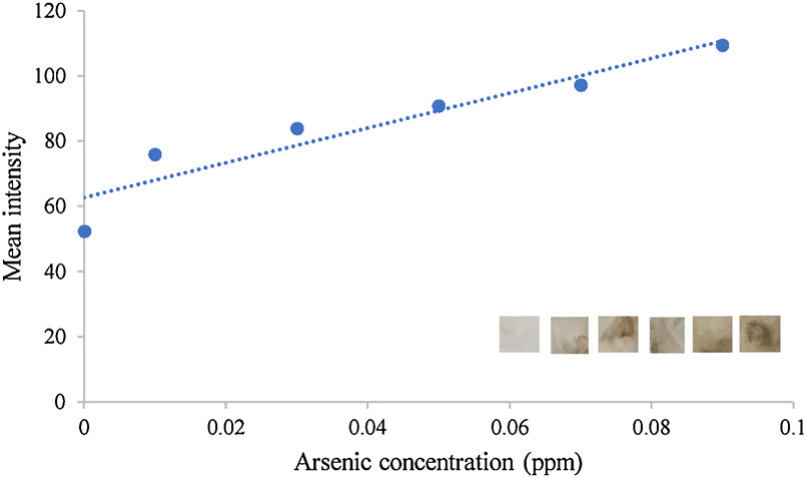
Graph of the mean color intensity vs arsenic concentration.

## Conflict of interest

The authors declare that there are no conflicts of interest.
